# Increased Risk of Hepatocellular Carcinoma Associated With Neighborhood Concentrated Disadvantage

**DOI:** 10.3389/fonc.2018.00375

**Published:** 2018-09-11

**Authors:** Denise Danos, Claudia Leonardi, Aubrey Gilliland, Sharmila Shankar, Rakesh K. Srivastava, Neal Simonsen, Tekeda Ferguson, Qingzhao Yu, Xiao-Cheng Wu, Richard Scribner

**Affiliations:** ^1^Louisiana State University Health Sciences Center School of Medicine, Stanley S. Scott Cancer Center, New Orleans, LA, United States; ^2^Behavioral and Community Health Sciences Department, Louisiana State University Health Sciences Center School of Public Health, New Orleans, LA, United States; ^3^Epidemiology Department, Louisiana State University Health Sciences Center School of Public Health, New Orleans, LA, United States; ^4^Department of Genetics, Louisiana State University Health Sciences Center School of Medicine, Stanley S. Scott Cancer Center, New Orleans, LA, United States; ^5^Biostatistics Department, Louisiana State University Health Sciences Center School of Public Health, New Orleans, LA, United States

**Keywords:** hepatocellular carcinoma, social determinants, neighborhood environment, multilevel analysis, neighborhood concentrated disadvantage

## Abstract

**Purpose:** Over the past three decades, Hepatocellular Carcinoma (HCC) is one of few cancers for which incidence has increased in the United States (US). It is likely social determinants at the population level are driving this increase. We designed a population-based study to explore whether social determinants at the neighborhood level are geographically associated with HCC incidence in Louisiana by examining the association of HCC incidence with neighborhood concentrated disadvantage.

**Methods:** Primary HCC cases diagnosed from 2008 to 2012 identified from the Louisiana Tumor Registry were geocoded to census tract of residence at the time of diagnosis. Neighborhood concentrated disadvantage index (CDI) for each census tract was calculated according to the PhenX Toolkit data protocol based on population and socioeconomic measures from the US Census. The incidence of HCC was modeled using multilevel binomial regression with individuals nested within neighborhoods.

**Results:** The study included 1,418 HCC cases. Incidence of HCC was greater among males than females and among black than white. In multilevel models controlling for age, race, and sex, neighborhood CDI was positively associated with the incidence of HCC. A one standard deviation increase in CDI was associated with a 22% increase in HCC risk [Risk Ratio (RR) = 1.22; 95% CI (1.15, 1.31)]. Adjusting for contextual effects of an individual's neighborhood reduced the disparity in HCC incidence.

**Conclusion:** Neighborhood concentrated disadvantage, a robust measure of an adverse social environment, was found to be a geographically associated with HCC incidence. Differential exposure to neighborhoods characterized by concentrated disadvantage partially explained the racial disparity in HCC for Louisiana. Our results suggest that increasing rates of HCC, and existing racial disparities for the disease, are partially explained by measures of an adverse social environment.

## Introduction

Over the past three decades hepatocellular carcinoma (HCC) is one of the few cancers for which incidence has increased in the United States (US) (1, 2). This is alarming because HCC has been recognized by the US Congress as a recalcitrant cancer of which the 5 year survival is less than 50%^1^. Consequently, it is projected that by 2030 cancers of the liver and bile duct will be the third leading cause of cancer deaths in the US (3). The increase has been primarily attributed to three risk factors leading to hepatic dysfunction: (1) Hepatitis C virus (HCV) infection; (2) obesity-related metabolic dysfunction leading to Non Alcoholic Fatty Liver Disease (NAFLD); and (3) alcohol-use disorders (AUD). While HCV infection represents the greatest individual risk among the three, what is driving the epidemic at the population level is more complex. Due to the high prevalence of metabolic syndrome (20%) and obesity (35%) in the general population, the population attributable fraction (PAF) for NAFLD is estimated at 32%, followed by HCV infection at 20.5% and AUD at 13.4% (4, 5). Consequently, it may be that the continued increase in HCC will be driven by the rising rates of obesity related metabolic disorders leading to NAFLD (6, 7).

Studies that have modeled and monitored the trend in HCC incidence suggest the peak in HCC will occur soon (8, 9). However, there is concern that these projected trends are primarily based on the risk of HCC associated with HCV infection and therefore too optimistic (6, 10, 11). Over the past decade, the incidence in HCV infection has been decreasing due to a cohort born between 1945 and 1964 that have had high rates of HCV infection, which is not evidenced in the birth cohorts born before 1945 or after 1964 (12). On the other hand, rates of HCC due to NAFLD are increasing dramatically both in the US and internationally due to the decades old obesity pandemic (11, 13, 14). The current study examines the role of a neighborhood risk factor, concentrated disadvantage, associated with both intravenous (IV) drug use risk and obesity risk to determine whether residence in a disadvantaged neighborhood explains both HCC incidence overall and disparities in HCC incidence by race. The study uses data from the Surveillance Epidemiology and End Results (SEER), Louisiana Tumor Registry for 2008–2012. It is hoped that by identifying modifiable factors in neighborhood environments effective intervention can be initiated to target the rising incidence of HCC in Louisiana.

## Materials and methods

### HCC case ascertainment

This study involved a secondary analysis of data from the Louisiana Tumor Registry (LTR) and US Census. LTR is a member of the of National Cancer Institute's (NCI) Surveillance, Epidemiology and End Results (SEER) Program, as well as a member of the Centers for Disease Control and Prevention's (CDC) National Program of Cancer Registries. Primary cases of HCC diagnosed from January 2008 to December 2012 were identified by International Classification of Diseases for Oncology, Third Edition (ICDO-3) site code C220 and histology codes 8170-8175 and 8180. The Louisiana State University Health Sciences Center-New Orleans Human Research Protection Program and Institutional Review Board reviewed and approved this research project.

Incident cases of HCC among adults 35 years and older were defined as individuals diagnosed with at least one case of primary invasive HCC during the study period. *In-situ* tumors were not included. Age was categorized into three age groups (35–49, 50–64, and 65 and older) (8). With this age grouping, individuals in the 50–64 age group for this study period (2008–2012) generally fall into the 1945–1965 birth cohort that experienced higher rates of HCV compared to birth cohorts before 1945 and after 1965. Sex was defined as male or female. Race was defined as black or white; we did not include other races in the study due to their small numbers. Thus, the total number of possible individual level demographic risk factor combinations for the study was 12.

### Geocoding cases and determining disadvantage

Individual HCC cases were geocoded to 2010 US census tracts using the Automated Geospatial Geocoding Interface Environment system, which was developed through a partnership between the North American Association of Central Cancer Registries (NAACCR), Texas A&M University, and the NCI as a single, uniform geocoding platform for open use by cancer registries^2^ Cases were geocoded to census tracts by street address at time of diagnosis, with 95% success rate. At-risk population for census tracts was determined by 2010 US Decennial Census data (15, 16).

Neighborhood concentrated disadvantage index (CDI) scores were calculated based on the PhenX Toolkit protocol. The PhenX Toolkit is a product from the collaboration between the Research Triangle Institute and the National Human Genome Research Institute to develop consensus measures for phenotypes and exposures^3^ CDI is a construct that operationalizes urban theory regarding the overconcentration of blacks, children and female-headed families in economically disadvantaged neighborhoods (17). We derived CDI using American Community Survey (ACS) 2008–2012 Five-year estimates for census tracts^4^ Tracts were scored through a principle components analysis of 6 measures (given as percentages): (1) individuals below the federal poverty line, (2) households receiving public assistance income, (3) female-headed households, (4) individuals that are unemployed, (5) individuals that are below the age of 18, and (6) individuals that are black. Factor scores for study census tracts follow a standard normal distribution with a mean of zero and standard deviation of 1.

### Census tract exclusions

According to the US Census, there were 1,148 census tracts in Louisiana in 2010. Standard US census tracts typically have between 2,500 and 8,000 residents and are relatively homogenous with respect to population characteristics, economic status, and living conditions^5^ We excluded 19 Louisiana census tracts with zero population in 2010 as well as 8 non-standard tracts with a population less than 500 people. Because census tracts were designed to contain relatively homogenous populations, we did not feel it was appropriate to merge the population for these tracts with neighboring tracts. We excluded a single census tract that encompasses Orleans Parish Prison. The prison had a population of 3,059 in 2010. No incident cases of HCC were omitted based on these tract exclusions. After these exclusions, individuals from 1,120 Louisiana census tracts remained eligible for the study. We further restricted the study area to urban parishes (counties), as we have used census tracts to define “neighborhoods” and this unit best represents neighborhoods for urban areas only. Of 64 Louisiana parishes (counties), 47 were classified as urban according to US Office of Management and Budget's definition of a metropolitan statistical area as “one or more adjacent counties or county equivalents that have at least one urban core area of at least 50,000 population, plus adjacent territory that has a high degree of social and economic integration with the core as measured by commuting ties”^6^ 1,038 out of the 1,120 previously identified census tracts were in urban parishes (counties). Urban tracts contained 93% of eligible population and 94% of the HCC cases identified.

### Population at risk aggregation

Based on the study design, cases of HCC were aggregated for each of the 12 demographic risk combinations in 1,038 study census tracts, which yielded 12,456 possible data points or “cells.” The population at risk for age, sex, and race specific cells within each census tract was determined from 2010 US census population counts, multiplied by 5 to represent person-years at risk. For each data cell, cancer cases and population-years were used to construct a binomial random variable, where the response was given as the number of incident HCC cases over the person-years at. A total of 196 data cells had no at risk population (person-years) and did not contribute to the analysis. There was a single incident case of HCC recorded for these cells but due to a lack of population at risk, the case was not included in the analysis.

### Statistical analysis

Multiple multilevel generalized linear regression models were used to model the incidence of HCC in Louisiana. The multilevel data structure consisted of individuals (level 1) nested within census tracts (level 2). The number of incident HCC cases over the population years at risk constituted a binomial random response. Models used a log link to estimate adjusted risk ratios for the study population. Correlation among individuals within the same tract was modeled with the use of a random intercept for each tract. All statistical analyses were performed with SAS version 9.4 (SAS Institute, Cary, NC). Multilevel generalized linear models were executed with the Glimmix Procedure, using maximum likelihood estimation based on adaptive quadrature rule. Model fit was assessed through the Pearson Chi-Square goodness of fit statistic (18).

An initial model was used to estimate individual demographic trends in HCC incidence and assess if there was clustering of cases at the neighborhood level. This model contained fixed effects for individual level demographic variables, age, sex and race. We also included effect modifiers (interactions) for the 50–64 age group to account for distinct patterns of HCC incidence in the HCV cohort. Model 1 was used to determine neighborhood variation in HCC incidence after accounting for the composition of individuals within census tracts. A second model included CDI in order to evaluate whether neighborhood disadvantage explained census tract variation in incidence and if CDI contributed to existing racial disparities. Model 3 included an effect modifier (interaction) for the effects of CDI in the 50–64 age group. A *p*-value smaller than 0.05 for two sided statistical tests was considered statistically significant.

## Results

Study population characteristics are presented as Table 1. The study included 2,057,053 Louisiana residents, of which 53% were female and 71% were white. There was a notable difference in neighborhood disadvantage for the population by race, with black residents disproportionately represented in more disadvantaged areas. The disparity in neighborhood living environment was evidenced by a greater mean CDI score for blacks [mean = 0.54, standard deviation (SD) = 9.60] compared to whites (mean = −0.49, SD = 9.33).

**Table 1:** Study population characteristics by race in urban parishes (counties) of Louisiana, 2010.

	**Total**	**White**	**Black**
*N*	2,057,053	1,460,131	596,922
**AGE (%)**			
35–49	38.08	36.45	42.05
50–64	37.64	37.26	38.57
65+	24.28	26.29	19.38
**SEX (%)**			
Female	52.92	52.00	55.20
Male	47.08	48.00	44.80
CDI, mean (SD)	−0.19 (11.21)	−0.49 (9.33)	0.54 (9.60)

*CDI, Concentrated Disadvantage Index; SD, Standard Deviation*.

**Figure 1:**
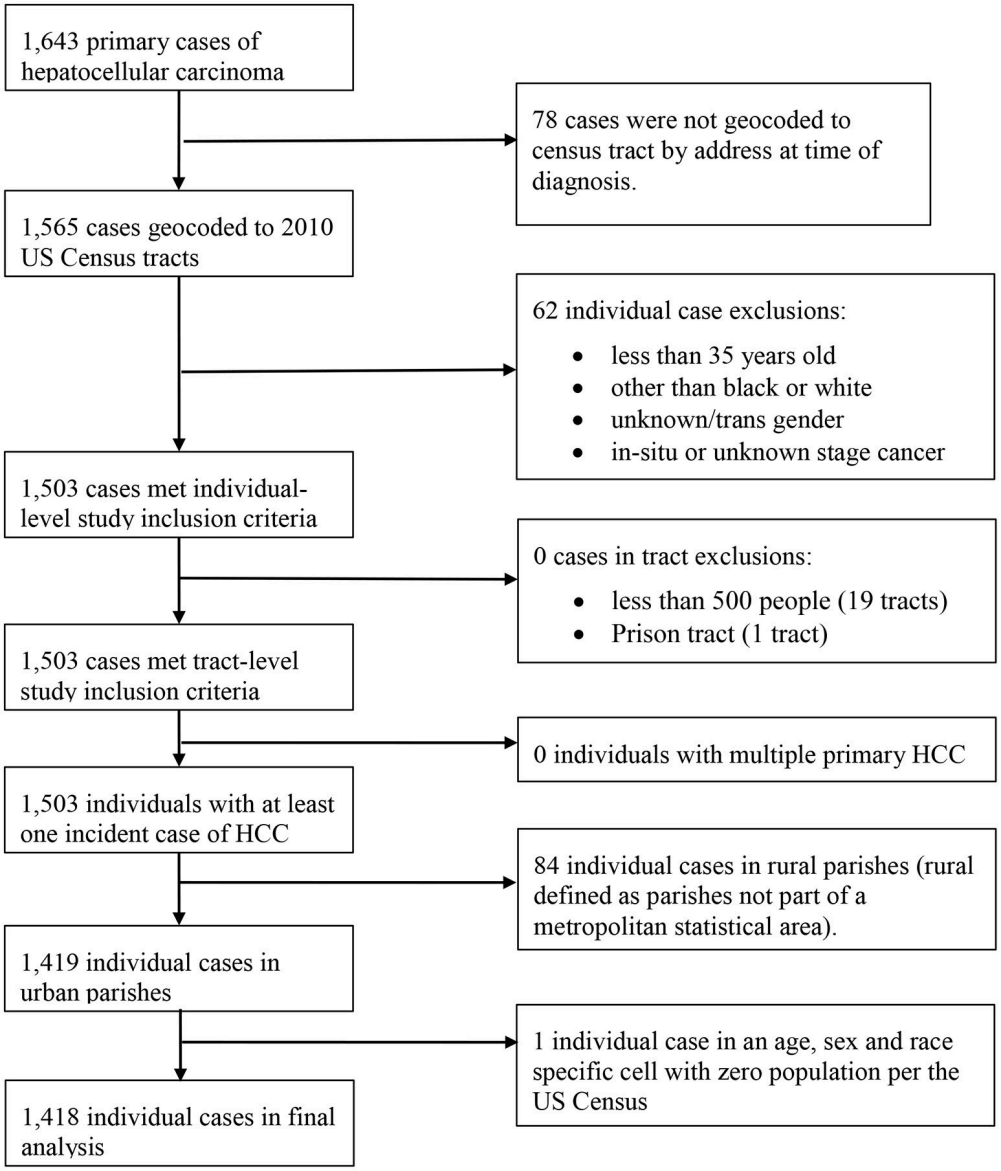
Case inclusion summary.

**Figure 2:**
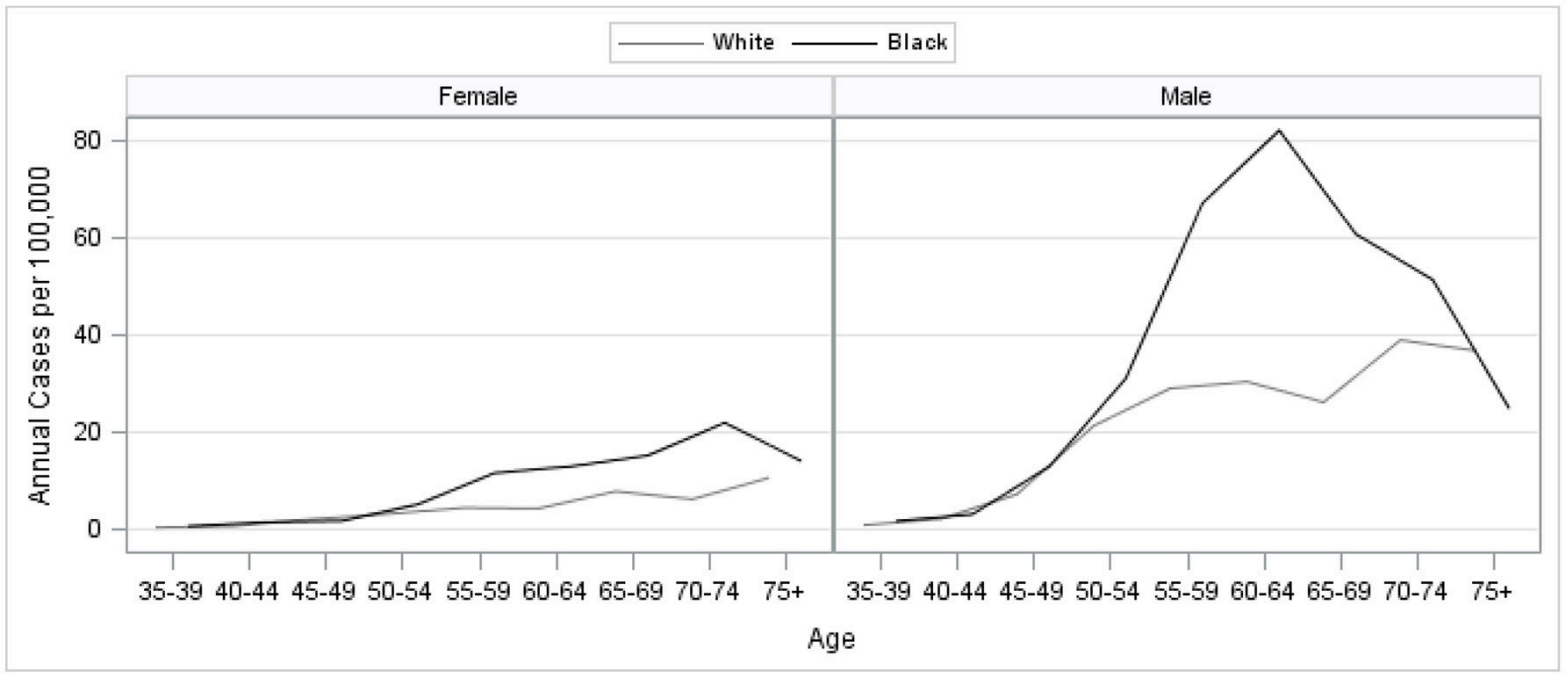
Age, sex, and race-specific HCC incidence, urban parishes (counties) of Louisiana 2008–2012.

We identified 1,418 incident cases of HCC in the study population that met the study criteria (Figure 1). Trends in HCC incidence by age group showed that HCC risk increased with advancing age, with the exception of a peak observed for ages 50–69, particularly among black males (Figure 2). Although we are only considering HCC, these trends are consistent with national trends found for liver and bile duct cancer during this time (2).

**Figure 3:**
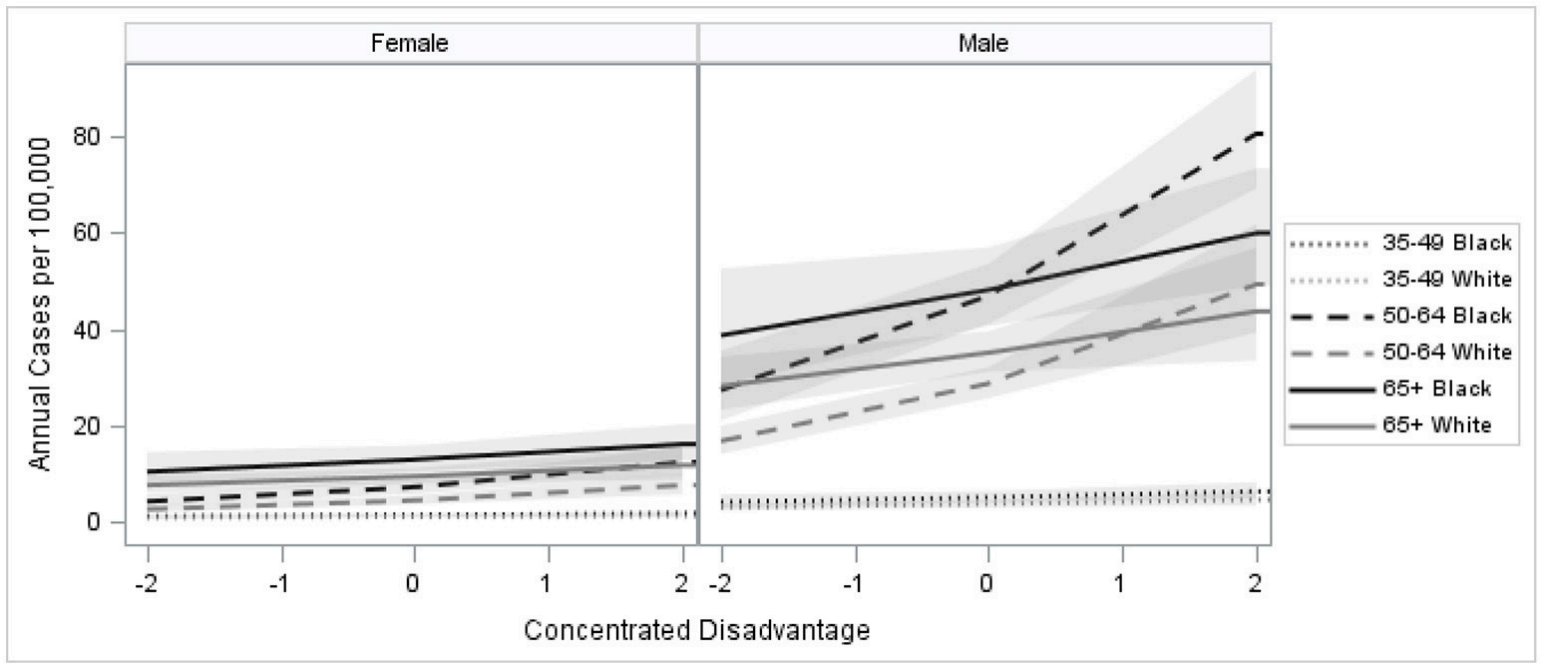
Age, sex, and race-specific predicted HCC risk by neighborhood concentrated disadvantage index, urban parishes (counties) of Louisiana 2008–2012.

Multivariable multilevel model parameter estimates are provided as Table 2. There was significant neighborhood variation in HCC incidence in the first model, which controlled for demographic composition (*p* = 0.0158). Neighborhood variation in HCC incidence was reduced after adjusting for the effects of neighborhood CDI (Models 2 and 3). Estimated relative risk (RR) and corresponding 95% confidence intervals (CI) are provided as Table 3. Results from Model 1 indicated that men were 3.72 times as likely to develop the disease compared to women (RR = 3.72; 95% CI: 3.13–4.43 and that risk was 1.53 times greater in black residents compared to whites (RR = 1.53; 95% CI: 1.29–1.80). Relative risk for the 50–64 age group, which contains the US HCV birth cohort, differed significantly from other age groups; men in this cohort had 6.50 times the risk of women (RR = 6.50; 95% CI: 5.31–7.96) and black residents had 2.18 times the risk of white residents (RR = 2.18; 95% CI: 1.89–2.52). Neighborhood CDI was significantly associated with HCC, with increased risk exhibited in more disadvantaged areas (Model 2). CDI is a sample-based index, where scores from a sample have a mean of 0 and a standard deviation of 1. Therefore, a single unit increase in CDI represents a one standard deviation increase in neighborhood disadvantage. Results from the second model indicate a single unit increase in CDI was associated with 22% relative increase in HCC risk (RR = 1.22, 95% CI: 1.15–1.31). Further, there was a significant interaction between CDI and age, where the effects of CDI were greater among the 50–64 age group (Model 3). In our final model, the adjusted risk ratio for a single unit increase in CDI was estimated to be 1.31 (RR = 1.31; 95% CI: 1.20–1.43) for the 50–64 age group while it was 1.12 (RR = 1.12; 95% CI: 1.01–1.23) among other age groups. Controlling for differential effects of neighborhood CDI on HCC incidence reduced the estimated relative risk for blacks to 1.37 (95% CI: 1.13–1.67). In the 50–64 age group, the estimated relative risk for blacks was reduced to 1.64 (95% CI: 1.37–1.95). The excess racial disparity for the 50–64 age group was no longer statistically significant from the other ages, as indicated by the overlap in the confidence intervals for the two estimates in this model. Estimated trends in HCC incidence from the final model are presented in Figure 3, where we observe the most profound effects of CDI in black males aged 50–64.

**Table 2:** Parameter estimates from multilevel binomial regression models of Hepatocellular Carcinoma incidence in urban parishes (counties) of Louisiana, 2008–2012.

	**Model 1**			**Model 2**			**Model 3**		
**Effect**	**Estimate**	**Std err**	***P*-value**	**Estimate**	**Std err**	***P*-value**	**Estimate**	**Std err**	***P*-value**
Intercept	−11.5902	0.1275	<0.0001	−11.4932	0.1282	<0.0001	−11.5344	0.1296	<0.0001
Age 50–64	1.4392	0.1625	<0.0001	1.4325	0.1625	<0.0001	1.5001	0.1649	<0.0001
Age 65+	2.2672	0.1087	<0.0001	2.2534	0.1087	<0.0001	2.2607	0.1087	<0.0001
Male	1.3145	0.0889	<0.0001	1.3204	0.0889	<0.0001	1.3172	0.0889	<0.0001
50–64^*^Male	0.5577	0.1361	<0.0001	0.5556	0.1361	<0.0001	0.5602	0.1362	<0.0001
Black	0.4230	0.0849	<0.0001	0.2112	0.0922	0.0220	0.3169	0.1010	0.0017
50–64^*^Black	0.3570	0.1109	0.0013	0.3611	0.1109	0.0011	0.1748	0.1339	0.1919
CDI				0.2017	0.0332	<0.0001	0.1096	0.0505	0.0303
50–64^*^CDI							0.1607	0.0653	0.0138
Tract Variance	0.0748	0.0348	0.0158	0.0543	0.0334	0.052	0.0536	0.0334	0.0539
χ^2^/df	1.07			1.08			1.07		

*CDI, Concentrated Disadvantage Index; All models include random intercept for US census tracts*.

**Table 3:** Adjusted risk ratios (RR) and 95% confidence intervals (CI) from multilevel binomial regression models of Hepatocellular Carcinoma incidence in urban parishes (counties) of Louisiana, 2008–2012.

	**Model 1**	**Model 2**	**Model 3**
	**RR (95% CI)**	**RR (95% CI)**	**RR (95% CI)**
**SEX**			
Female (ref)	1.00	1.00	1.00
Male at age group 1	3.72 (3.13, 4.43)	3.74 (3.15, 4.46)	3.73 (3.14, 4.44)
Male at age group 2	6.50 (5.31, 7.96)	6.53 (5.33, 7.99)	6.54 (5.34, 8.00)
**RACE**			
White (ref)	1.00	1.00	1.00
Black at age group 1	1.53 (1.29, 1.80)	1.24 (1.03, 1.48)	1.37 (1.13, 1.67)
Black at age group 2	2.18 (1.89, 2.52)	1.77 (1.51, 2.08)	1.64 (1.37, 1.95)
CDI, 1 SD increase		1.22 (1.15, 1.31)	
CDI, 1 SD increase at age group 1			1.12 (1.01, 1.23)
CDI, 1 SD increase at age group 2			1.31 (1.20, 1.43)

*CDI, Concentrated Disadvantage Index; SD, Standard Deviation*.

*All models control for age, sex and race. Adjusted risk ratios for sex, race and CDI are provided separately for age group 1 (35–49 years old and 65 and over) from age group 2 (50–64 years old). All models include random intercept for US census tracts*.

## Discussion

The current study assessed individual demographic and neighborhood level risk in HCC incidence. We found that neighborhood concentrated disadvantage (CDI), a robust measure of an adverse social and physical environment, was strongly associated with increased HCC risk, with 22% relative increase in risk associated with a single standard deviation increase in disadvantage. These results parallel those from an investigation using data from the NIH-AARP study, which found an association between area socioeconomic deprivation and increased risk of HCC incidence (19). We found that disparities in HCC by race and sex were significantly greater in the 50-64 age group. This age group includes the majority of a 1945–1965 birth cohort with elevated rates of HCV. Thus, disparities measured through population age-adjusted HCC incidence rates have been apparently influenced by HCV risk in this birth cohort. For our study population, there was differential exposure to neighborhood concentrated disadvantage by race, and we found that adjusting for the contextual effects of CDI reduced the observed racial disparities in HCC for all age groups. Additionally, our results show the effects of CDI on HCC were more pronounced in the 50-64 age group that correlates with the US HCV cohort compared to other ages (RR = 1.31 vs. 1.12), which is consistent with the established role of HCV infections as a key mechanism in HCC risk.

Given the observed geographic association between neighborhood disadvantage and HCC incidence, which apparently accounted for some of the racial disparity in HCC incidence, it is worth discussing how neighborhood disadvantage relates to each of the major risk factors for HCC. Not surprisingly, there is literature linking each of these risk factors to neighborhood disadvantage.

IV drug use has been the main driver of HCV in the US (20, 21). Disadvantaged neighborhoods have long been linked to IV drug use. Galea and colleagues indicate neighborhood disadvantage is a central component in their framework of contextual determinants of IV drug use (22). Neighborhood disadvantage has been linked to younger age of initiation, greater injection frequency, cessation failure, and unsafe syringe use among IV drug users (23–27). While we found the effect of neighborhood disadvantage was greater in the 50–64 year old age group, corresponding to the birth cohort at greatest risk of IV drug use and therefore HCV risk, it should be noted that CDI was still strongly associated with HCC risk in other age groups. This is consistent with the epidemiology that indicates obesity related NAFLD has the largest population attributable fraction for HCC.

It is widely conceded that local obesogenic environments are responsible for the obesity epidemic (28). Given the dramatic increase in obesity rates over the past decades, it is the role of these local obesogenic environments that is most concerning with regard to increasing HCC incidence. Disadvantaged populations are widely acknowledged to live in more obesogenic environments. In fact, there are a number of reviews suggesting that the disparities in obesity related outcomes like NAFLD are due to these neighborhood effects that disproportionately affect poor and minority populations (29–36). It is interesting to note that although neighborhood environments are implicated, the specific physical or social conditions responsible for the increased obesity risk has yet to be agreed upon (37, 38). Consequently, attempts to link specific elements of neighborhood obesogenic environments to HCC incidence beyond neighborhood disadvantage would represent a significant contribution to this literature.

Finally, alcohol abuse also tends to be associated with disadvantaged neighborhoods. Overconcentration of alcohol outlets at the neighborhood level historically has been linked to alcohol abuse (39–41). In addition, alcohol outlets tend to be disproportionately located in poor and minority neighborhoods despite the fact that higher SES and white race is associated with higher levels of alcohol use (42). However, the fact that levels of alcohol use have been stable over the period does not suggest overconcentration of alcohol outlets at the neighborhood level are driving the increase in HCC incidence.

It is vital to determine the underlying mechanisms of HCC risk. Successful antiviral treatment has been shown to reduce the progression of liver disease and the development of HCC in HCV patients at all stages of liver disease (43, 44). The effort to address the high rate of HCV is directed at the 1945–1965 birth cohort (45). As this birth cohort ages and antiviral treatment rates improve, obesity related NAFLD will take over as the predominant risk factor for HCC (11). Although several studies have reported significant protective effects of certain medications used to manage chronic metabolic conditions associated with NAFLD (i.e., metformin for diabetes, statins, aspirin) (11, 46), research is needed to identify the neighborhood factors driving the obesity epidemic in order to develop effective preventive strategies targeting disadvantaged neighborhood.

From a physiologic perspective, the link between obesity related NAFLD and HCC is extremely relevant in terms of identifying the neighborhood level risk factor. Epidemiological data suggest the association of about 15% of human cancers with chronic infection and inflammation (47). Inflammation plays a key role in the pathogenesis of chronic liver injury and is considered to be a risk factor for HCC (48). About 90% of incident HCC develops due to chronic liver inflammation, the induction of fibrosis and subsequent cirrhosis (49). Inflammation is involved in cell degeneration, fibrosis, cirrhosis and tumor formation, which are essential stages of HCC development. During HCC initiation, events such as mutations, deletions or overexpression of genes provide mutant cells with a growth and survival advantage (50). However, recent studies demonstrate that these initial genetic alterations or epigenetic changes are not sufficient for a complete neoplastic progression, suggesting that HCC initiation and progression might depend on consistently supportive signals that are provided from an inflammatory microenvironment thus facilitating each step of carcinogenesis (51, 52).

Limitations of this investigation stem from to the cross-sectional nature of the study design. The duration of exposure or the risk associated with neighborhood environment over time is unable to be established, as is temporality between exposure and incident HCC. CDI was measured at time of HCC diagnosis, yet etiologic exposure occurs much earlier in the natural history of this cancer. The study lacks data on individual level risk factors such as HCV infection, NAFLD, and alcohol use that are needed to determine the distribution of underlying clinical risks of this disease. An additional limitation is that we have assessed the effects of neighborhood living environment based on a census-defined spatial unit (tract), which is designed to be relatively homogenous in terms of social characteristics but does lack a subjective definition of “neighborhood.”

The mobility of residents who live within these neighborhoods included in the present study also poses another limitation. As an investigation regarding cancer incidence, it is understood the environmental influence upon the development of HCC began many years before diagnosis. Therefore, the possibility exists that study participants resided within a neighborhood with more disadvantage and then moved into a less disadvantaged neighborhood recorded upon HCC diagnosis. With this, literature describing residential mobility and health have found there is a lack of effect on cross-sectional studies (53). Movers select for neighborhoods of similar health, especially among those with poorer health (54, 55).

The generalizability of this study is also limited when reviewing the racial makeup of the study population. It has been shown Hispanics are experiencing the fastest increase in HCC incidence, with a 35.8% increase between 2003 and 2011 (56). However, only blacks and whites were included in our analyses due to sample size; Louisiana has a very small Hispanic population, with Hispanic individuals encompassing only 3.9% of Louisiana's population of 4.4 million in 2010^7^ In a study utilizing Texas Cancer Registry data, rates of HCC in Hispanics were estimated to be 3–4 times that of non-Hispanic whites (57). While incidence rates of HCC differ by ethnicity, trends in population attributable fraction of incident HCC associated with NAFLD in the Hispanic population are similar to those in non-Hispanic whites (39.3 and 34.8%, respectively). Conversely, blacks show a greater population attributable fraction of HCC associated with HCV (36.1%), with only 14.4% of incident HCC with an etiologic cause of NAFLD (4). The population attributable fraction of incident HCC varies widely by race, and the influence of neighborhood on this variation should be further investigated in the future.

Through employing multilevel analyses this study has identified a statistically significant contribution of neighborhood concentrated disadvantage to racial disparities in HCC in Louisiana. Our results suggest that increasing rates of HCC, and existing racial disparities in the disease, are partially driven by social contexts of adverse living conditions. Our group is continuing to investigate quantifying additional physical and social environment variables to include in subsequent analysis of the neighborhood environment to risk of HCC. Future studies in this area should also investigate whether associations between neighborhood environment and incident HCC are mediated by clinical manifestations of the primary risk factors for HCC.

## Author contributions

DD, CL contributed to the study concept, design, methods, and manuscript development. AG made contributions of intellectual content and manuscript development. SS, RS made contributions of intellectual content. NS, TF revised the article for intellectual content. QY, X-CW contributed to documentation of data and methods and have revised the article for intellectual content. RS made substantial contributions to the study concept, design and intellectual content. All authors have approved the content and agree to be accountable for the work with regard to accuracy and integrity.

### Conflict of interest statement

The authors declare that the research was conducted in the absence of any commercial or financial relationships that could be construed as a potential conflict of interest.

## Footnotes

^1^https://www.congress.gov/bill/112th-congress/house-bill/733.

^2^NAACCR Geocoder https://www.naaccr.org/gis-resources/.

^3^PhenX Toolkit – Neighborhood Concentrated Disadvantage Index https://www.phenxtoolkit.org/index.php?pageLink=browse.protocoldetails&id=211302.

^4^American FactFinder https://factfinder.census.gov/faces/nav/jsf/pages/index.xhtml.

^5^US Census Geographic Areas Reference Manual https://www.census.gov/geo/reference/garm.html.

^6^2010 Office of Management and Budget (OMB) Standards https://www.census.gov/programs-surveys/metro-micro/about/omb-standards.html.

^7^2010 US Census https://www.census.gov/2010census/.
